# Normative Values for Self-Reported Benchmark Workout Scores in CrossFit® Practitioners

**DOI:** 10.1186/s40798-018-0156-x

**Published:** 2018-08-20

**Authors:** Gerald T. Mangine, Brant Cebulla, Yuri Feito

**Affiliations:** 1 Exercise Science and Sport Management, Kennesaw State University, 520 Parliament Garden Way NW, Kennesaw, GA 30144 USA; 2 Hass School of Business, University of California at Berkeley, Berkeley, CA USA

**Keywords:** Fitness assessment, Sport-specific, Athlete classification, High-intensity functional training

## Abstract

**Background:**

CrossFit® practitioners commonly track progress by monitoring their ability to complete a variety of standardized benchmark workouts within a typical class setting. However, objective assessment of progress is challenging because normative data does not currently exist for any of these benchmark workouts. Therefore, the purpose of this study was to develop normative values for five common benchmark workouts (i.e., Fran, Grace, Helen, Filthy-50 [F50], and Fight-Gone-Bad [FGB]).

**Methods:**

Performance data from 133,857 male (_M_) and female (_F_) profiles located on a publicly available website were collected and sorted by sex (i.e., male [_M_] and female [_F_]) and competitive age classification (i.e., teen [T], individual [I], or masters [M]) and screened for errors. Subsequently, 10,000 valid profiles were randomly selected for analysis.

**Results:**

Means and standard deviations were calculated for each category for Fran (I_M_ 250 ± 106 s; I_F_ 331 ± 181 s; M_M_ 311 ± 138 s; M_F_ 368 ± 138 s; T_M_ 316 ± 136 s; and T_F_ 334 ± 120 s), Grace (I_M_ 180 ± 90 s; I_F_ 213 ± 96 s; M_M_ 213 ± 93 s; M_F_ 238 ± 100 s; T_M_ 228 ± 63 s; and T_F_ 223 ± 69 s), Helen (I_M_ 9.5 ± 1.9 min; I_F_ 11.1 ± 2.4 min; M_M_ 10.2 ± 2.0 min; M_F_ 11.5 ± 2.3 min; T_M_ 9.4 ± 1.6 min; and T_F_ 12.7 ± 1.9 min), F50 (I_M_ 24.4 ± 5.9 min; I_F_ 27.3 ± 6.9 min; M_M_ 26.7 ± 6.1 min; M_F_ 28.2 ± 6.0 min; T_M_ 25.9 ± 7.9 min; and T_F_ 28.3 ± 8.1 min), and FGB (I_M_ 335 ± 65 repetitions; I_F_ 292 ± 62 repetitions; M_M_ 311 ± 59 repetitions; M_F_ 280 ± 54 repetitions; T_M_ 279 ± 44 repetitions; and T_F_ 238 ± 35 repetitions). These values were then used to calculate normative percentile (in deciles) values for each category within each workout. Separate, one-way analyses of variance revealed significant (*p* < 0.05) differences between categories for each workout.

**Conclusions:**

These normative values can be used to assess proficiency and sport-specific progress, establish realistic training goals, and for standard inclusion/exclusion criteria for future research in CrossFit® practitioners.

## Key Points

- Normative scores for five common benchmark workouts (i.e., Fran, Grace, Helen, Filthy-50, and Fight-Gone-Bad) were created for male and female competitors in the teen, individual, and masters’ competitive age divisions for CrossFit®.
- On average, males in the individual and masters’ age categories scored better than their female counterparts in each workout despite workouts being scaled for sex.
- The normative scores reported here may be used for standardized comparison between athletes, to track individual progress, and as an inclusionary/exclusionary criteria tool for future investigations on CrossFit®.

## Background

CrossFit® training combines weightlifting, gymnastics, and traditional cardiovascular exercise modalities (e.g., running, rowing, and cycling) into a single workout that is performed at high intensity. Daily workouts are generally unique and vary in the number and type of exercises included, the prescribed intensity and volume loads, and whether rest intervals are enforced (e.g., Fight-Gone-Bad [FGB] requires a 1-min rest break between rounds) [1]. Performance during such workouts may be quantified through a variety of strategies. Trainees may be instructed to complete all exercises and/or rounds as quickly as possible, they may be asked to complete “as many repetitions as possible” (AMRAP) within a certain time frame, or they may be asked to maintain a specific workout pace (e.g., complete a specified number of repetitions every minute) for a set time frame. Regardless of formatting, workouts will typically challenge some combination of strength, power, endurance, and/or sport-specific skill. While monitoring progress in attributes such as strength, power, and endurance may be accomplished via traditional laboratory and field assessments, monitoring progress in sport-specific skill is not as simple. Assessments of individual skills (e.g., rope jumping or climbing, bar and ring muscle-ups, burpees, and box jumps) may provide some insight, but this practice lacks context. To this end, common benchmark workouts (i.e., FGB, Fran, Grace, Helen, and Filthy-50 [F50]) may be used to assist practitioners in gauging their ability to perform various movements within the context of a workout. Currently, normative values exist for several traditional physiological measures (e.g., maximal strength, aerobic capacity) [2], but not for these common benchmark workouts.

The CrossFit® website allows users to create a profile where they can upload their best scores for traditional measures of strength (i.e., squat, deadlift), power (i.e., clean and jerk, snatch), anaerobic performance (i.e., 400-m sprint), aerobic performance (i.e., 5000-m run), and common benchmark workouts. Previously, proficiency in some of these benchmark workouts (i.e., Fran and Grace) have been related to anaerobic performance and strength [3], while self-reported performances may distinguish competitive level within this sport [4]. For instance, Serafini and colleagues (2017) noted that performances in common benchmark workouts were greater in higher-ranking male and female competitors who placed within the top 1500 during the 2016 CrossFit® Open (CFO). However, considering that over 320,000 individuals participated in the 2016 CFO [5], this information is limited to a relatively small sample of CrossFit® practitioners, and only to those associated with the most competitive division (i.e., individual). Therefore, the purpose of this investigation was to create normative values for the five common benchmark workouts across the three primary competitive age divisions (i.e., individual, masters, and teens) in CrossFit® practitioners.

## Methods

### Study Design

Five-hundred thousand uniform resource locators (URL) were scraped (May 25–August 14, 2017) from a publicly available online database [6] and yielded 133,857 user profiles that contained self-reported anthropometric and performance data. Profiles were sorted by sex and competitive age classification (i.e., individual, masters, or teens) and then screened for errors. Profiles were eliminated from the analysis if they (a) contained data points that exceeded four standard deviations (i.e., < 0.001% of all cases) from their respective mean [7] or (b) did not contain more than one completed benchmark workout (i.e., Fran, Grace, Helen, Filthy-50, and Fight-Gone-Bad). Of the remaining cases (*n* = 39,884), exactly 10,000 profiles were randomly selected for analysis.

### Participants

Male (_M_) and female (_F_) participants, who were assigned to the individual (I; 18–34 years), masters (M; ≥ 35 years), or teens (T; < 18 years) age-classifications during the 2017 CFO, were selected for this study. All participants possessed, of their own volition and initiative, a profile on the CrossFit Games™ website [6] where their self-reported performance data was located. Profiles were selected by the numerical order of their URL. All data was downloaded from The CrossFit Games™ website and decoded so that no identifiable information (i.e., name) was available from any of the participants. Random sampling of all valid cases elicited 4397 profiles in I_M_ (30.0 ± 4.2 years; 178.8 ± 7.2 cm; 86.3 ± 10.6 kg), 1628 profiles in I_F_ (29.9 ± 4.0 years; 164.5 ± 6.7 cm; 65.2 ± 8.5 kg), 2955 profiles in M_M_ (42.0 ± 5.9 years; 178.9 ± 7.1 cm; 87.3 ± 11.2 kg), 918 profiles in M_F_ (41.7 ± 5.9 years; 164.7 ± 6.7 cm; 64.7 ± 9.0 kg), 69 profiles in T_M_ (17.5 ± 2.7 years; 175.3 ± 6.5 cm; 74.5 ± 10.3 kg), and 33 profiles in T_F_ (17.0 ± 0.8 years; 163.4 ± 6.5 cm; 61.4 ± 8.8 kg). Since these data were pre-existing and publicly available, the University’s Institutional Review Board classified this study as exempt (Study# 16-215).

### Performance Measures

Participants have the option on their profile to record their best performances for select benchmark workouts. These include Fight-Gone-Bad (FGB), Fran, Grace, Helen, and the Filthy 50 (F50). The details of each workout’s design, repetition scheme, exercise list, standardized load or difficulty, and scoring method are described in Table 1. Briefly, four of the recorded events (i.e., Fran, Grace, Helen, and F50) were scored by time-to-completion (TTC), while FGB was scored as the total number of repetitions completed within the set time frame.

**Table 1:** Description of standards for five common benchmark workouts

Name	Workout	Repetitions scheme	Exercises	Male standards	Female standards	Scoring
Fran	3 consecutive rounds	Round 1 = 21 repetitions	•Thrusters	43.1 kg	24.5 kg	TTC (± 0.1 s)
		Round 2 = 15 repetitions	•Pull-ups			
		Round 3 = 9 repetitions				
Filthy 50	1 continuous circuit	50 repetitions for each exercise	•Box jumps	0.6 m box		TTC (± 0.1 min)
			•Jumping pull-ups	Bar height set at a height equal to mid-forearm of fully extended shoulders and elbows		
			•“American” kettlebell swings	16.4 kg		
			•Walking lunges			
			•Knees-to-elbows			
			•Push press	20.4 kg	13.6 kg	
			•Back extensions			
			•Wall-ball shots	9.1 kg, 3 m target	6.4 kg, 2.7 m target	
			•Burpees			
			•Double-unders			
Helen	1 continuous circuit completed three times in succession	400 m	•Sprint			TTC (± 0.1 min)
		21 repetitions	•“American” kettlebell swings	24.0 kg		
		12 repetitions	•Pull-ups			
Grace	1 round	30 repetitions	•Clean and jerks	61.2 kg	43.1 kg	TTC (± 0.1 s)
Fight Gone Bad	3 rounds of five 1-min stations with each round separated by a 1-min rest period	AMRAP	•Wall-ball shots	9.1 kg, 3 m Target	6.4 kg, 2.7 m Target	Total repetitions
			•Sumo deadlift high-pull	34.0 kg	24.9 kg	
			•Box jumps	0.5 m box		
			•Push press	34.0 kg	24.9 kg	
		1 cal = 1 repetition	•Ergometer rowing for calories			

*AMRAP* as many repetitions as possible, *TTC* time to completion

### Statistical Analyses

Statistical software (SPSS, v.24.0, SPSS Inc., Chicago, IL) was used for random sampling, as well as to calculate means, standard deviations, and percentiles (in deciles) for each competitive group. Additionally, a one-way analysis of variance was used to examine differences between I_M_, I_F_, M_M_, M_F_, T_M_, and T_F_. Subsequent Tukey’s post hoc tests were used to determine pairwise differences when significant *F* ratios were obtained. For all statistical tests, a probability level of *p* ≤ 0.05 was established to denote statistical significance.

## Results

The percentile ranking scores for all competitive groups are presented in Table 2. Significant differences were found between age-classification and sex groupings for FGB (*F* = 100.2, *p* < 0.001), Fran (*F* = 168.5, *p* < 0.001), Grace (*F* = 71.3, *p* < 0.001), Helen (*F* = 142.7, *p* < 0.001), and F50 (*F* = 38.2, *p* < 0.001).

**Table 2:** Percentile ranking scores for competitive age classifications and sex in common benchmark workouts

	90th	80th	70th	60th	50th	40th	30th	20th	10th
Fight Gone Bad (repetitions)									
Individual men	418	387	369	352	335	319	301	284	252
Individual women	371	341	324	308	292	276	260	243	213
Masters’ men	387	358	342	326	311	296	280	264	235
Masters’ women	349	323	308	293	280	266	251	237	210
Teen boys	335	314	302	290	279	268	257	245	223
Teen girls	282	265	256	246	238	229	219	210	193
Fran (seconds)									
Individual men	114	166	195	223	250	277	306	334	386
Individual women	98	187	236	284	331	377	425	474	563
Masters’ men	134	201	239	276	311	346	383	421	488
Masters’ women	191	259	296	333	368	403	440	477	545
Teen boys	140	207	244	281	316	350	387	424	491
Teen girls	179	238	271	303	334	364	397	429	488
Grace (seconds)									
Individual men	64	108	133	157	180	203	227	252	296
Individual women	89	136	163	188	213	238	263	290	337
Masters’ men	93	139	164	189	213	237	261	287	332
Masters’ women	109	158	185	212	238	264	291	318	367
Teen boys	147	178	195	212	228	244	261	279	310
Teen girls	134	168	186	205	223	240	259	277	311
Helen (minutes)									
Individual men	7.98	9.16	9.82	10.46	11.08	11.69	12.34	12.99	14.17
Individual women	7.68	8.64	9.18	9.71	10.21	10.72	11.24	11.78	12.75
Masters’ men	8.60	9.71	10.33	10.94	11.52	12.10	12.72	13.33	14.45
Masters’ women	7.39	8.16	8.58	9.00	9.39	9.79	10.21	10.63	11.39
Teen boys	7.39	8.16	8.58	9.00	9.39	9.79	10.21	10.63	11.39
Teen girls	10.21	11.14	11.66	12.17	12.66	13.15	13.66	14.18	15.11
Filthy 50 (minutes)									
Individual men	16.76	19.66	21.27	22.86	24.37	25.88	27.47	29.08	31.98
Individual women	18.49	21.86	23.73	25.57	27.33	29.08	30.92	32.79	36.17
Masters’ men	18.80	21.81	23.49	25.13	26.70	28.27	29.91	31.58	34.60
Masters’ women	20.43	23.38	25.02	26.63	28.17	29.70	31.32	32.95	35.91
Teen boys	15.65	19.54	21.71	23.84	25.86	27.89	30.02	32.18	36.08
Teen girls	17.88	21.85	24.06	26.23	28.30	30.37	32.54	34.75	38.72

### Fight Gone Bad

**Fig. 1:**
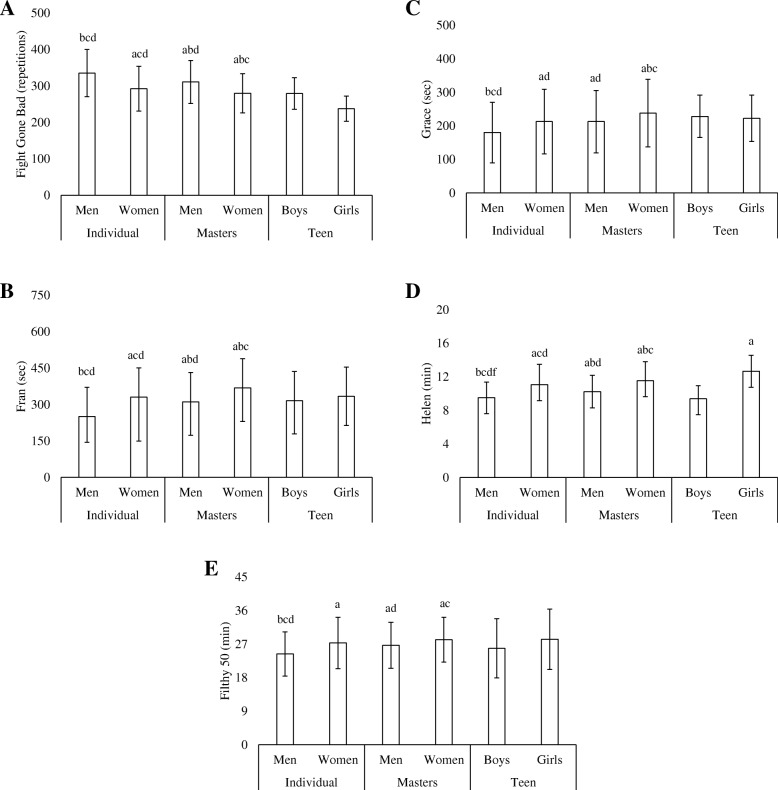
Comparisons between competitive age-classifications and sex in common benchmark workouts (**A**. Fight Gone Bad; **B**. Fran; **C**. Grace; **D**. Helen; **E**. Filthy 50). **a**. Significantly (*p* < 0.05) different from individual men. **b**. Significantly (*p* < 0.05) different from individual women. **c**. Significantly (*p* < 0.05) different from master’s men. **d**. Significantly (*p* < 0.05) different from master’s women. **e**. Significantly (*p* < 0.05) different from teen boys. **f**. Significantly (*p* < 0.05) different from teen girls

For FGB (Fig. 1a), I_M_ (335 ± 65 repetitions) reported completing more (*p* < 0.001) repetitions than I_F_ (292 ± 62 repetitions), M_M_ (311 ± 59 repetitions), and M_F_ (280 ± 54 repetitions). M_M_ reported completing more (*p* < 0.001) repetitions than I_F_ and M_F_, while I_F_ reported completing more repetitions than M_F_ (*p* = 0.005). No differences were observed between teen competitors (T_M_ = 279 ± 44 repetitions; T_F_ = 238 ± 35 repetitions) and any other classification.

### Fran

For Fran (Fig. 1b), I_M_ (250 ± 106 s) reported completing the workout faster (*p* < 0.001) than I_F_ (331 ± 181 s), M_M_ (311 ± 138 s), and M_F_ (368 ± 138 s). M_M_ reported completing Fran faster (*p* < 0.001) than I_F_ and M_F_, while I_F_ reported faster completion times than M_F_ (*p* < 0.001). No differences were observed between teen competitors (T_M_ = 316 ± 136 s; T_F_ = 334 ± 120 s) and any other classification.

### Grace

For Grace (Fig. 1c), I_M_ (180 ± 90 s) reported completing the workout faster (*p* < 0.001) than I_F_ (213 ± 96 s), M_M_ (213 ± 93 s), and M_F_ (238 ± 100 s). Both I_F_ and M_M_ reported completing the workout faster (*p* < 0.001) than M_F_. No differences were observed between teen competitors (T_M_ = 228 ± 63 s; T_F_ = 223 ± 69 s) and any other classification.

### Helen

For Helen (Fig. 1d), I_M_ (9.51 ± 1.87 min) reported completing the workout faster (*p* < 0.001) than I_F_ (11.08 ± 2.41 min), M_M_ (10.21 ± 1.97 min), M_F_ (11.52 ± 2.28 min), and T_F_ (12.66 ± 1.91 min). M_M_ reported completing Helen faster (*p* < 0.001) than I_F_ and M_F_, while I_F_ reported faster completion times than M_F_ (*p* ≤ 0.001). No other differences were observed between T_M_ (9.4 ± 1.6 min) and any other classification.

### Filthy 50

For F50 (Fig. 1e), I_M_ (24.37 ± 5.92 min) reported completing the workout faster (*p* < 0.001) than I_F_ (27.33 ± 6.88 min), M_M_ (26.7 ± 6.15 min), and M_F_ (28.17 ± 6.02 s), while M_M_ reported completing the workout faster than M_F_ (*p* < 0.001). No differences were observed between teen competitors (T_M_ = 25.9 ± 7.9 min; T_F_ = 28.3 ± 8.1 min) and any other classification.

## Discussion

CrossFit® training constantly varies daily workouts to promote general physical preparedness [1]. While this strategy appears to be useful for eliciting adaptations across a variety of fitness domains [8, 9], gauging sport-specific progress and proficiency is difficult. Traditional field and laboratory measures (e.g., aerobic capacity, anaerobic threshold, peak power) are commonly accepted tools for monitoring athletic progress [2], and a few have been related to CrossFit® performance [3, 10]. However, in most instances, their precision is dependent on the availability of expensive equipment, and it may not be logistically feasible to assess several individuals from a single location or across locations, without sacrificing their validity and/or reliability. It is also difficult to simulate actual workouts or competitive environments with traditional assessment tools (e.g., metabolic cart, cycle ergometers, force plates) because of the likelihood that they would impair natural movement. Thus, CrossFit® practitioners commonly use standardized workouts to monitor sport-specific adaptations. These common benchmark workouts are identifiable by name (e.g., Fran, Grace), and their requirements are standardized across affiliates. Though commonly practiced, there is little information available to allow practitioners to determine the quality of their performance in such workouts. Here, we provide normative values for self-reported performance scores in five, common benchmark workouts for male and female practitioners across the three, primary age-classifications (i.e., teens, individuals, or master’s) of the CrossFit® Open. Practitioners can use these data to project their status among their peers, as well as to monitor their individual progress and set realistic goals for training.

In terms of absolute intensity, CrossFit® workouts prescribed for I_M_ are the most challenging. For instance, in the workouts examined in the present study, men were typically required to lift more weight, jump onto a higher box, or throw a heavier medicine ball to a higher target than women. Workout prescription may be further scaled to accommodate less experienced and/or older individuals, but this does not occur in the common benchmark workouts (i.e., only one workout design exists for each sex, regardless of age). Accordingly, we observed that I_M_ and I_F_ performed better than their master’s counterparts in all workouts aside from F50 (i.e., no differences were found between I_F_ and M_F_). This is not surprising because younger practitioners would be expected to perform better when given the same task [11, 12]. However, within the individual and master’s age classifications, men reported better scores than women for each workout. This is interesting because appropriate scaling should equate workout difficulty and result in similar scores between men and women. Typically, clear differences exist between men and women when comparisons are made with absolute values for traditional measures of strength and endurance, but not when using relative figures (e.g., percentage of one-repetition maximum, per kilogram of body mass) [13–15]. Though comparisons between sexes are not common in CrossFit®, it may be possible if relative standards are used when prescribing intensity. Another possible explanation may be related to the fact that more men (*n* = 7352) than women (*n* = 2546), in the individual and master’s age classifications, possessed a profile account and reported their performance scores. Likewise, only 102 teenage practitioners possessed an account in the present sample. Individuals who participate in CrossFit® and similar exercise forms are not required to create a profile on the CrossFit® website and have alternative platforms for tracking progress (e.g., Wodify, Zen Planner, beyond the whiteboard). Consequently, our findings may be limited to CrossFit® athletes who also possess an account on the CrossFit® website. Further, because the athletes report these data as their personal best performance in each workout, our findings may be most representative of peak fitness within each individual workout and not necessarily of ability across all workouts simultaneously.

These data may also be useful for developing more accurate inclusion/exclusion criteria in research. Currently, physiological research on CrossFit® is limited, and most studies have used training experience (i.e., the number of years of participation) as the primary indicator for training status. Though years of experience would likely indicate a degree of familiarity with the nuances of this training strategy, its use as an indicator of proficiency is complicated by individual variability in training frequency, regularity in utilizing prescribed (versus scaled) workouts, athletic talent, and previous experiences in other sports. Put simply, unless potential participants are recruited from a pool of individuals who have been previously ranked in international competitions (e.g., the Reebok CrossFit Games™), it is difficult to accurately identify their proficiency in the sport from experience alone. For instance, male and female participants have been previously recruited based on their experience (number of years was not reported) with CrossFit® to determine their physiological responses to two common benchmark workouts (including “Fran”) [16]. However, it may not be correct to extrapolate their findings to all CrossFit® practitioners. Based on our findings, the “Fran” scores for male (331 ± 82.4 s) and female (331 ± 92.1 s) participants in that study would have placed them within the 20th and 50th percentiles, respectively. It may have been more appropriate to describe those individuals as beginner or intermediate CrossFit® practitioners, rather than simply stating they had experience. Likewise, Butcher and colleagues (2015) recruited participants who had previously progressed to the regional round of the Reebok CrossFit Games™ or at least participated in the CrossFit® Open, and who possessed at least 1 year of experience (~ 3.7–4.3 years). However, by examining their measured performances in Fran (203 ± 48 s; range = 130–289 s) and Grace (136 ± 32 s; range = 93–194 s), and depending on sex category (not specified), they could have ranked above the 70th percentile for “Fran” or as low as the 20th percentile for “Grace”. Comparatively, less variability in reported performance scores can be observed in the study conducted by Serafini and colleagues (2017). In that study, the authors utilized final rankings in the 2016 CrossFit® Open to examine differences in benchmark workout scores reported by the top 1500 male and 1500 female athletes (i.e., the top ~ 1%). Although the reported scores would still vary by specific workout and sex, male and female participants typically ranked above the 80th and 70th percentiles, respectively. As more research is conducted on CrossFit®, it will become increasingly necessary to utilize more specific methods for participant recruitment to make accurate inferences across studies.

## Conclusions

In practice, the five benchmark workouts described here are typically made part of regular training but are not commonly completed under the scrutiny of a judge. Although it is possible that the self-reported data used in this study included invalid performance scores (i.e., the athlete did not meet all workout requirements), this method of reporting is consistent with how these workouts are commonly scored at a local affiliate. That is, coaches rely on trainees to follow the described standards for each workout and to accurately report their scores. Nevertheless, additional steps were taken in to minimize the number of unrealistic performances (i.e., removing scores that were greater than four standard deviations from the mean). Though potentially limited to users of the CrossFit® website, the normative values we have presented appear to adequately describe sport-specific ability for five common benchmark workouts. Practitioners and coaches may use these values to assess individual progress, make comparisons between individuals, and establish realistic training goals. Further, as more research is conducted on this training strategy, these values may be used as inclusion/exclusion criteria to assist researchers when assessing the suitability of potential participants for a study’s specific aims. Nevertheless, it may be worthwhile to verify these normative values, obtained from self-reported performance scores, with those obtained from observed performances. Additionally, the five workouts examined here represent a small sample of potential benchmark tools that could be used to assess sport-specific ability in CrossFit® participants. Future endeavors should seek to identify normative values for additional benchmark workouts (e.g., “Cindy”, “Jackie”, “Diane”), as well as for “Hero” workouts (e.g., “Jerry”, “Murph”, “Randy”).

## Abbreviations

AMRAP: As many repetitions as possible
_F_: Female
F50: Filthy-50
FGB: Fight-Gone-Bad
I: Individual competitive division (18–34 years)
_M_: Male
M: Master’s competitive division (≥ 35 years)
T: Teen competitive division (< 18 years)
TTC: Time-to-completion
URL: Uniform resource locator
